# H1299R in coagulation Factor V and Glu429Ala in MTHFR genes in recurrent pregnancy loss in Sari, Mazandaran

**Published:** 2016-05

**Authors:** Nadia Arabkhazaeli, Kasra Ghanaat, Mohammad Bagher Hashemi-Soteh

**Affiliations:** 1 *Department of Genetic, Faculty of Science, Damghan Branch, Islamic Azad University, Damghan, Iran.*; 2 *Department of Clinical Biochemistry and Genetics, Faculty of Medicine, Mazandaran University of Medical Sciences, Sari, Iran.*; 3 *Immunogenetic Research Center, Molecular and Cell biology Research Center, Faculty of Medicine, Mazandaran University of Medical Sciences, Sari, Iran. *

**Keywords:** *Spontaneous Abortion*, *Polymorphism (Genetics)*, *Factor V*, *Methylene tetra hydrofolate Reductase (MTHFR)*

## Abstract

**Background:**

Recurrent pregnancy loss (RPL) is caused by different factors, including genetics and thrombophilia. Beside Factor V Leiden, another nucleotide change in a factor V (FV) gene (A4070G; His1299Arg) has been identified linking to hereditary thrombophilia. Also, two proposed MTHFR polymorphisms, C677T and A1298C (Glu429A) are linked with RPL.

**Objective:**

In this study, the effect of two factors, A4070G in FV and A1298C in MTHFR are evaluated in RPL patients from Mazandaran province, Iran.

**Materials and methods:**

Sample population of 100 women with RPL and 100 controls with Mazandarani ethnics from northern Iran were consist. The factor V (A4070G) and MTHFR (A1298C) polymorphisms were genotyped by PCR-RFLP.

**Results:**

Molecular study showed 5 women from patients and 9 women from control group were heterozygous AG for A4070G. Frequency of "A" allele in patient and control groups was 97.5% (0.975) and 95.5% (0.955) respectively, and "G" allele frequency was 2.5% (0.025) and 4.5% (0.045) respectively. No significant association (p≤0.05) between FV A4070G genotype and RPL with an OR=1.88, CI 95%=0.6-5.82, was observed (p=0.4). Also, for A1298C, all patients and control individuals were AA genotype. "A" allele frequency in patients and control was 100% and "C" allele frequency was zero. There was no significant difference for A1298C between groups.

**Conclusion:**

Our finding showed that A4070G and A1298C polymorphisms cannot be considered as a cause of PRL in women from Mazandaran province, northern Iran.

## Introduction

Recurrent pregnancy loss (RPL) appears a signiﬁcant clinical problem affecting approximately 2% of women (1). RPL pathophysiology is poorly understood. Different factors, including genetic, immune defect, infection, anatomical as well as thrombophilia have been presumed as RPL causes (2, 3). Many recent studies have examined the mutations incidence in speciﬁc thrombophilia factor genes in women with unexplained pregnancy loss (4, 5). 

Mutations in coagulation factor V gene are among the most common causes for venous thrombosis and also for pregnancy complications, such as RPLs. One of the well-known mutation in factor V gene is (A1691G; R506Q). FV is inactivated by protein C (APC), so the amount and activity of FV is regulated by APC protein (6). Factor V Leiden mutation causes APC resistance which is associated with increased risk of venous thromboembolism (VTE) and RPL (7). In 1996, another nucleotide change in exon 13 of FV gene (A4070G; His1299Arg), known as R2, has identified and linked to hereditary thrombophilia (8). Previous studies have shown that His1299Arg change will increase 2-3 folds the venous thromboembolism (VTE) risk (8-10).

The MTHFR enzyme plays important roles in folates metabolism (11). In the MTHFR enzyme, C677T (rs1801133) and A1298C (rs1801131) single-nucleotide polymorphisms are two most important polymorphisms that affect folate and total homocysteine status. The MTHFRC677T, a cytosine (C) to a thymine (T) substitution at position 677 (Ala222Val), causes impaired folate binding and reduced activity of the MTHFR enzyme (12, 13). The activity of MTHFR enzyme is reduced by 35% in people who are 677CT carriers and by 70% among 677TT carriers, while the effect of A1298C polymorphism has not been demonstrated consistently (14). Two MTHFR polymorphic variants, C677T and A1298C (Glu429A) were analyzed in association with different disorders such as cancer, vascular disease, neural tube defects, hyperhomocysteinemia, as well as RPL (3, 12, 15-18).

The prevalence of these mutations varies among different populations and ethnic groups. To date, no comprehensive study has established on the relationship between H1299R and A1298C and RPL in Iranian populations. Present study tried to evaluate the association between H1299R and A1298C polymorphisms in Mazandarani ethnic's women with RPL from northern Iran. 

**Materials and methods**

**Subjects**

This case-control study was performed in Sari, capital city of Mazandaran province during the January 2013 to December 2013. Prior to enrollment, all patients were given an explanation of study nature, and written informed consent was obtained from all individuals. The study was approved by the Research Ethics Committee at the Islamic Azad University, Damghan Branch. 

RPL was defined as two or more spontaneous consecutive abortions at 5-20 weeks of gestation. The miscarriage history of women with RPL was examined and cases with anatomic, chromosomal, hormonal, autoimmune or infectious causes were excluded from this study. The study comprised of 100 women with RPL aged 20-45 years and 100 healthy fertile controls aged 27-44 years. 

**Genotyping of the Factor V A4070G variant**

**Figure 1:**
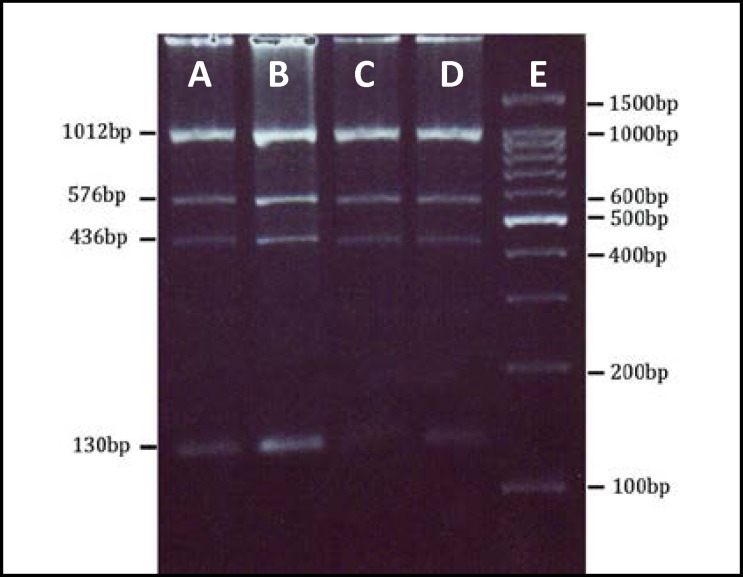
PCR-RFLP for factor V A4070G Polymorphism using *RsaI *restriction enzyme digestion. A3% agarose gel electrophoresis showed, while lane 1 represents AA genotype, lane 2 and 3 represent heterozygous (A/G) samples and lane 4 is an undigested sample with 1142 bp PCR product. Lane 5 shows a 100 bp plus DNA marker

The primers for the PCR reaction to analyze the A4070G (H1299R) polymorphism were: forward (5-GCA GAC AGT CAT CTC TCC AGA CCT-3) and reverse (5-CTC TGG AGG AGT TGA TGT TTG TCC-3) as previously described (19). PCR conditions included one step initial denaturation (94^o^C for 3 min), 35 cycles (94^o^C for 45 sec, 63.6^o^C for 40 sec and 72^o^C for 40 sec) and a final extension at 72^o^C for 5 min. PCR products were then electrophoresed in a 1.5% agarose gel (Fermentas, Germany). Amplified PCR products were subjected to enzymatic digestion with *RsaI* restriction enzyme (Fermentas, Germany) for 16 hr at 37^o^C and visualized after separation by 3% agarose gel electrophoresis followed by staining with ethidium bromide (Figure 1).

**Genotyping of the MTHFR A1298C variant**

**Figure 2:**
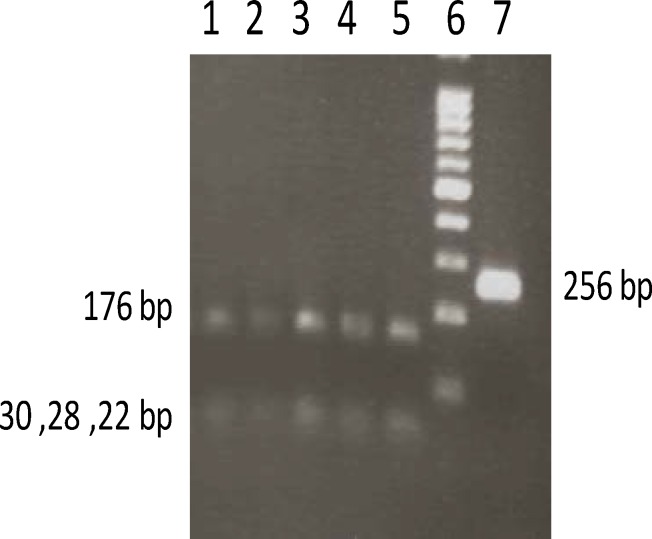
PCR-RFLP for MTHFR A1298C Polymorphism using *SatI *restriction enzyme digestion. Lane 1 to 5 represent AA genotypes that a 256 bp fragment digest to 176 and 30,28 and 22 bp fragments, respectively, while lane 7 shows an undigested PCR product. Lane 6 shows a 100 bp DNA marker on a 3% agarose gel electrophoresis

Venous blood collected from all participants was used to isolate genomic DNA restriction fragment length polymorphism analysis of polymerase chain reaction amplified fragments (PCR-RFLP) as previously described (20). Briefly, for the A1298C polymorphism, the primers were: forward (5-CTTCTACCTGAAGAGCAAGTC-3) and reverse (5-CATGTCCACAGCATGGAG-3), amplifying a 256 bp fragment as previously described (21). PCR conditions were included one step initial denaturation at 93^o^C for 3 min, followed by 35 cycles (94^o^C for 50 sec, 61^o^C for 40 sec and 72^o^C for 40 sec) and a final extension at 72^o^C for 5 min. Amplified PCR products were subjected to enzymatic digestion with *Sat1 (Fnu4h1)* for 16 hr at 37^o^C and visualized after separation by 3% agarose gel electrophoresis followed by staining with ethidium bromide (Figure 2).

**Statistical analysis**

Statistical analysis was performed using SPSS software version 16 and was analyzed using descriptive statistics and  ^2^ test. Statistical significance was set at p<0.05.

## Results

**Frequencies of** **Factor V A4070G polymorphism**

The VA4070G polymorphism was determined based on product size-band. The PCR amplification of *FV gene* including A4070G produced a 1142 bp fragment followed by *RsaI* restriction digestion. Enzyme digestion creates two fragments, 1012 bp and 130 bp in normal alleles (A), and three fragments, 130bp, 436bp and 576bp in mutant alleles (G) respectively (Figure 1). The genotypic and allelic frequencies of factor V A4070G polymorphisms in women with RPL and control group are demonstrated in table I. According to table I, the genotypic frequencies of factor V A4070Gin cases were 95% for AA, 5% for AG, without any GG genotype, and 91% for AA, 5% for AG and none for GG in control group respectively. These results showed that 5 women from cases and 9 women from control group were heterozygous for A4070G of factor V. The frequencies of A allele between RPL and control groups were 97.5% (0.975) and 95.5% (0.955) respectively. Also the G allele frequencies in these groups were observed 2.5% (0.025) and 4.5% (0.045) respectively. Statistical analysis showed no significant difference between two groups (p=0.4) (Table I).

**Frequencies A1298C polymorphism**

The PCR product for MTHFR gene A1298C polymorphism was 256 bp. Four pieces, including 22 bp, 28 bp, 30 bp and 176 bp were created after digestion with *SatI* (*Fnu4h1*) restriction enzyme in normal alleles (A), but three pieces, including 22 bp, 30 bp and 204 bp were expected in mutant alleles (C) respectively (Figure 2). The genotypic frequency of MTHFR gene A1298C was 100% for AA and no (0%) frequency was achieved for AC or CC genotypes in cases. Also, 100% frequency was seen for AA genotype and none (0%) for AC or CC for control group respectively. The frequency of A allele in A1298C polymorphism among the women with RPL and controls was1.0 and 0.0 for C respectively (Table I).

**Table I:** Genotype and allele frequency of factor V A4070G and MTHFR A1298C polymorphisms in patients and controls women from Mazandaran province northern Iran (n=100 in each group

			**Case N (%)**	**Control N (%)**	**OR (95% CI)**	**p-value**
A4070G						
	AA		95 (95%)	91 (91%)	1.88 (0.607 – 5.82)	0.407
	AG		5 (5%)	9 (9%)		
	GG		0 (0%)	0 (0%)		
	A		195 (97.5%)	191 (95.5%)		
	G		5 (2.5%)	9 (4.5%)		
A1298C					1.87(.84-4.17)	0.169
		AA	100	100		
		AC	0	0		
		CC	0	0		
		A	200 (100%)	200 (100%)		
		C	0 (0%)	0 (0%)		

For A4070G, the frequency of A allele between RPL and control groups were 97.5% (0.975) and 95.5% (0.955) respectively. Also the frequencies of the G allele in these two groups were observed 2.5% (0.025) and 4.5% (0.045) respectively. Statistical analysis showed no significant difference (p≤0.05) between two groups (p=0.4)

## Discussion

RPL is a multifactorial process and thrombophilic defect (22). This study was designed to determine the association between two polymorphisms in *F V and MTHFR *genes and RPL for the first time in Mazandaran province, northern Iran. Several investigations have reported an association between the MTHFR A1298C and Factor V H1299R (A4070G) polymorphisms with recurrent spontaneous abortion, whereas some studies have rejected this correlation (23-26).

Factor V has three polymorphisms, G1691A, A4070G (H1299R) and A5279G. Beside G1691A, factor V Leiden, several studies have shown that A4070G polymorphism also could cause thrombophilia and play a role in coagulation factor V deficiency, but factor V Leiden (G1691A) is well known as a risk factor. Although Zammiti *et al* have rejected the association between Factor V H1299R (A4070G) polymorphisms with recurrent spontaneous abortion, but they verified the correlation between homozygousity of G/G in A4070G with increased risk of recurrent abortion after 8 wks of pregnancy (p>0.0002) (25, 26). 

In current study, the frequency of heterozygous AG genotype for factor V A4070G was observed 5% in patients and 9% in control groups, that have not shown significantly association with recurrent abortion (p=0.267). Also low level frequency of G allele in patients (2.5%) and controls (4.5%) (Table I) showed no statistically difference between patients and controls in women from northern Iran. These results are consistent with the results achieved by Coulam et al that rejected the association between A4070G with recurrent miscarriage (27).

A1298C polymorphism in the MTHFR gene is believed that changes the MTHFR enzyme activity (28). The Effect of C677T polymorphism in MTHFR enzyme activity was already studied and reported (27, 29, 30), however, a few studies have been done on A1298C polymorphism in our population to date. It already has shown that hyperhomocysteinemia and homozygous for MTHFR gene polymorphism, are risk factors for spontaneous abortion, whereas some researchers have rejected the relation between MTHFR polymorphisms and recurrent miscarriage (31-33). 

Wang *et al* indicated that A1298C polymorphism is not significantly different between RPL and control group, but AA genotype frequencies among women with RPL is significantly lower than control group (34). In one study, Mtiraoui *et al* reported that the prevalence of AAin A1298C in RPL group was significantly higher than the control, but in three separate studies, Hohlagschwandtner *et al,* Khaleghparast *et al, *and Poursadegh *et al* showed no relationship between C677T or A1298C polymorphisms in MTHFR with recurrent spontaneous abortion (35-38). Khaleghparast *et al* in a similar study in Iran reported that frequencies of AA genotype in A1298C MTHFR were 100% and the frequencies of AC or CC genotype were achieved zero (37). 

## Conclusion

Our results indicate that the MTHFR A1298C and Factor V A4070G polymorphisms are not significantly correlated with RPL in our population, women from Mazandaran province, northern Iran.

## Note

***This article extracted from M.Sc. thesis. (Nadia Arabkhazaeli)***
